# A novel laccase from *Trametes polyzona* with high performance in the decolorization of textile dyes

**DOI:** 10.1186/s13568-024-01687-3

**Published:** 2024-03-20

**Authors:** Daniela Bucchieri, Marco Mangiagalli, Francesca Martani, Pietro Butti, Marina Lotti, Immacolata Serra, Paola Branduardi

**Affiliations:** 1 Department of Biotechnology and Biosciences, University of Milano-Bicocca, Piazza Della Scienza 2, 20126 Milano, Italy; 2 Department of Material Science and Nanotechnology, CORIMAV Program, University of Milano-Bicocca, Via R. Cozzi 55, 20125 Milano, Italy

**Keywords:** *Trametes polyzona*, Laccase, Laccase mediator system, Acetosyringone, White-rot fungi, Textile dyes

## Abstract

Laccases are multicopper oxidases able to oxidize several phenolic compounds and find application in numerous industrial applications. Among laccase producers, white-rot fungi represent a valuable source of multiple isoforms and isoenzymes of these multicopper oxidases. Here we describe the identification, biochemical characterization, and application of laccase 2 from *Trametes polyzona* (TP-Lac2)*,* a basidiomycete fungus emerged among others that have been screened by plate assay. This enzyme has an optimal temperature of 50 °C and in acidic conditions it is able to oxidize both phenolic and non-phenolic compounds. The ability of TP-Lac2 to decolorize textile dyes was tested in the presence of natural and synthetic mediators at 30 °C and 50 °C. Our results indicate that TP-Lac2 most efficiently decolorizes (decolorization rate > 75%) malachite green oxalate, orange G, amido black10B and bromocresol purple in the presence of acetosyringone and 2,2′-azinobis (3-ethylbenzthiazoline-6-sulfonate)—ABTS. Overall, the laccase mediator system consisting of TP-Lac2 and the natural mediator acetosyringone has potential as an environmentally friendly alternative for wastewater treatment in the textile industry.

**Supplementary Information:**

The online version contains supplementary material available at 10.1186/s13568-024-01687-3.

## Key points

A novel laccase from *T. polyzona* was induced by the addition of veratryl alcohol

TP-Lac2 was heterologous expressed in *S. cerevisiae* and biochemically characterized

TP-Lac2 efficiently decolorizes textile dyes using acetosyringone as mediator

**Supplementary Information:**

The online version contains supplementary material available at 10.1186/s13568-024-01687-3.

## Introduction

In the textile industry, dyeing operations require a large number of dyes of different chemical classes generating polluting wastewater. Some of these dyes are recalcitrant to biodegradation, mutagenic and/or carcinogenic, and if released into the aquatic environment, might affect algal growth (Pearce 2003; Forgacs et al. 2004). Textile effluent treatments rely mainly on physical and chemical procedures, which are quite expensive and sometimes produce hazardous by-products (Shindhal et al. 2021). The use of microorganisms or their enzymes in textile wastewater treatment is of great interest in the development of sustainable dye degradation processes (Sarkar et al. 2017). Among enzymes, lignin-modifying enzymes (such as laccases, manganese and lignin peroxidases) have been proven to effectively oxidize synthetic dyes (Bilal et al. 2017; Kumar and Chandra 2020).

Laccases belong to the multi-copper oxidase family and are composed of three cupredoxin-like domains, which have the typical Greek key β-barrel fold (Piontek et al. 2002). The active site of laccases generally contains four copper ions coordinated by the T1, T2 and T3 centers (Piontek et al. 2002; Jones and Solomon 2015). The mononuclear T1 center, which has the highest redox potential, oxidizes the substrate and transfers electrons to the trinuclear center, formed by the T2 and T3 centers, which reduces oxygen to water (Piontek et al. 2002; Jones and Solomon 2015). Laccases are able to catalyze the oxidation of a diverse array of chemical compounds including polyphenols, diamines and aromatic amines and inorganic ions. Consequently, they are employed in different processes including lignin valorisation, (bio) polymers degradation and water bioremediation (Riva 2006; Ostadhadi-Dehkordi et al. 2012; Rovaletti et al. 2023). Promoting the advancement of more sustainable processes involves expanding the range of substrates and applications for laccases, for instance, by combining these enzymes with aromatic molecules, creating the so-called laccase/mediator system (LMS) (Riva 2006; Morozova et al. 2007). In LMS, the mediator undergoes initial oxidation by laccase, producing a highly oxidizing compound capable of subsequently oxidising the target substrate (Riva 2006; Morozova et al. 2007). Although the most commonly used mediators are of synthetic origin, such as 2,2′-azinobis(3-ethylbenzthiazoline–6-sulfonate) (ABTS) and 2-(2-hydroxyphenyl) benzothiazole (HBT), the current trend emphasizes the imperative shift toward natural mediators like syringaldehyde (SA), vanillic acid (VAc), and acetosyringone (AS) (Camarero et al. 2005; Cañas and Camarero 2010). AS and SA are phenolic compounds deriving from syringin, a glycoside present in several plants (Park et al. 1996; Baker et al. 2005; Cañas and Camarero 2010), while VAc is a dihydroxybenzoic acid deriving from vanillin (Lesage-Meessen et al. 1996; Converti et al. 2010). These natural mediators can be obtained from the processing of lignin (Camarero et al. 2005; Ganewatta et al. 2019) or via microbial bioconversion as in the case of vanillin (Lesage-Meessen et al. 1996; Converti et al. 2010).

In nature, the white-rot fungi are the primary producers of laccases, which are involved in several fungal physiological processes including for instance sporulation, stress defence and lignin degradation (Riley et al. 2014; Martani et al. 2017). To enhance their catalytic potential, some white-rot fungi possess multiple genes coding for laccase, the expression of which is finely regulated by species-dependent mechanisms (Palmieri et al. 2000). Generally, white-rot fungi have the ability to synthesize multiple isoforms of laccases during submerged cultivation and to enhance their synthesis in the presence of inducers. Conversely, certain fungi only produce laccases when exposed to certain inducers (Martani et al. 2017). The various isoforms have different biochemical properties in terms of optimal catalysis conditions, substrate specificity and stability (Mansur et al. 1998; Michniewicz et al. 2006; Ramírez-Cavazos et al. 2014). The number and type of isoforms produced by white-rot fungi depends on several factors, including growth conditions and the inducer used (Martani et al. 2017). The mechanisms underlying the expression of laccase genes are poorly investigated to date; for instance in some white-rot fungi the expression of certain laccase isoforms is regulated by a CreA-like glucose-binding protein (Mansur et al. 1998; Myasoedova et al. 2015). In these fungi, the laccase activity was observed when the glucose was completely depleted from the culture medium (Mansur et al. 1998; Myasoedova et al. 2015). This lack of knowledge might result in the production of natural enzymatic cocktail which might represent the desired product. Nonetheless, when one specific enzyme is desired, or should be better characterised for specific properties, the heterologous expression in a microorganism naturally not producing laccase is the choice of election.

Among white-rot fungi, *Trametes polyzona,* a basidiomycete fungus belonging to the *Polyporaceae* family, is known for producing several lignin degradation enzymes including the partially characterized isoform 1 (TP-Lac1) (Pi̇Nar et al. 2017). In this work, laccase 2 from *T. polyzona* has been identified, heterologously produced in *Saccharomyces cerevisiae*, biochemically characterized and tested for its ability to degrade textile dyes. TP-Lac2 shares 77% of sequence identity with TP-Lac1 and differs from it in some biochemical properties including optimum temperature of catalysis and substrate specificity. The ability of TP-Lac2 to degrade synthetic dyes was tested in the presence of different synthetic and natural mediators. Our results show that this enzyme exhibits highest performances in decolorization (> 75%) in the presence of ABTS and AS.

## Materials and methods

### General

Dyes including: amido black 10B, orange G, bromocresol purple sodium salts and malachite green oxalate were purchased from Carl Roth (Karlsruhe, Germany). Erythrosin B was purchased from Merck KGaA (Darmstadt, Germany) as well as the laccase mediators ABTS, HBT, AS, VAc and SA. The Bradford reagent used in this experiment was acquired from Bio Rad (Hercules, CA, USA). Yeast extract and peptone were provided by Biolife Italiana S.r.l. (Milan, Italy). All the other reagents were purchased from Merck KGaA (Darmstadt, Germany) unless stated otherwise.

### Strains and culture conditions

*T. polyzona* (MUCL 38443), *Deadaleopsis confragosa* (MUCL 28241), *Heterobasidion annosum* (MUCL 6136) *Stereum hirsutum* (MUCL 32895) and *Stereum ostrea* (MUCL 33680) were purchased at BCCM (Belgian Coordinated Collections of Microorganisms, Brussels, Belgium) and *Armillaria mellea* (DSM 1654) was purchased from the Leibniz Institute DSMZ (German Collection of Microorganisms and Cell Cultures GmbH, Braunschweig, Germany). They were grown on malt extract agar (malt extract 30 g/L, peptone 5 g/L and agar 15 g/L-MEA) at 30 °C. The strains were periodically transferred on fresh MEA and stored at 4 °C.

*Escherichia coli* strain DH5*ɑ* was stored at − 80 °C in cryotubes with 50% (v/v) glycerol. *E. coli* cells were used for cloning, propagating and storing procedures and they were cultured at 37 °C in lysogeny broth (LB) medium (NaCl10 g/L, peptone10 g/L  and  yeast extract 5 g/L ) supplemented with 100 µg/mL of ampicillin, when necessary.

*S. cerevisiae* strain CEN.PK 102-5B (*MATa*; *ura3–52*; *his3–11*; *leu2–3/112*; *TRP1*; *MAL2–8c*; *SUC2*) was obtained from Dr. P. Kötter (Institute of Microbiology, Johann Wolfgang Goethe-University, Frankfurt, Germany), grown on YPD medium (glucose 20 g/L, peptone 20 g/L and yeast extract 10 g/L) and stored in cryotubes at − 80 °C with 20% glycerol. All the liquid cultivations of *S. cerevisiae* strain CEN.PK 102-5B were conducted in shake flasks at 30 °C and on the 3400RVT orbital shaker (Cavallo s.r.l, Buccinasco, Italy) at 160 rpm with a ratio of flask volume:medium of 5:1.

### Production of putative laccase enzymes from white-rot fungi

To further investigate the laccase production, the selected white-rot fungi that caused the most significant green color development of the surrounding media were sub-cultured on MEA plates. Once the mycelium completely covered the plate, the entire colony was harvested using a sterile cotton swab and inoculated in synthetic liquid medium (MM—glucose 10 g/L, yeast extract 2 g/L, KH_2_PO_4_ 1 g/L, Na_2_HPO_4_ 0.2 g/L, MgSO_4_∙7H_2_O 0.5 g/L, C_4_H_4_Na_2_O_6_ 0.4 g/L, FeSO_4_∙7H_2_O 7 mg/L, ZnSO_4_∙7H_2_O 4.6 mg/L, MnSO_4_∙H_2_O 3.5 mg/L, CuSO_4_ ∙5H_2_O 0.7 mg/L, EDTA 50 mg/L, pH 6.0 (adjusted with 2 M KOH)). All submerged cultures were incubated for 20 days in the absence and in the presence of 10 mM veratryl alcohol (VA). Samples were withdrawn from flasks every 48 h, filtered, and centrifuged at 2000 g for 10 min and the supernatants (100 µL) were tested for laccase activity.

### Laccase activity assay

Laccase activity assays were performed using 0.5 mM ABTS as the reducing substrate in 50 mM sodium tartrate buffer at pH 4.0 and at room temperature. The increases in absorbance at 420 nm (ɛ_420_ = 36 mM^−1^∙cm^−1^) was measured in a Jasco V770 UV/NIR spectrophotometer (Jasco Europe, Cremella, Italy). One unit of enzyme activity is defined as the amount of enzyme that oxidizes 1 µmol of substrate *per* minute under assay conditions (Litwińska et al. 2019).

### Isolation, cloning and expression of the laccase gene TP-Lac2 in *S. cerevisiae*

After 9 days of cultivation in presence of 10 mM VA, the total RNA of *T. polyzona* was extracted using the ZR Fungal/Bacterial RNA MiniPrep^™^ Kit (ZYMO RESEARCH Freiburg im Breisgau, Germany) and reverse transcribed by ProtoScript^®^_®_ II First Strand cDNA Synthesis Kit (New England Biolabs^®^ Inc., Ipswich, MA, USA). The laccase sequence was amplified with primers containing the *Eco*RI restriction site and designed on the laccase sequence from *T. polyzona* laccase (TP-Lac1) available in the NCBI database (NCBI: KT802746, Table 1). Reactions were carried out using a Q5^®^ High-Fidelity DNA Polymerase (New England Biolabs^®^ Inc., Ipswich, MA, USA) under the following conditions: 1 cycle (98 °C for 5 min), 30 cycles (98 °C 1 min, 60 °C 1 min, and 72 °C 1 min), and a final cycle of 72 °C for 5 min. The amplified DNA was bi-directionally sequenced and cloned between *Eco*RI sites into the yeast shuttle expression vector pSAL4 under the control of copper-inducible *CUP1* promoter. pSAL4 contains the *S. cerevisiae* 2 micron plasmid and *E. coli* f1 origins of replication, the ampicillin resistance and the *URA3* selection markers (Zentella et al. 1999). *S. cerevisiae* strain CEN.PK 102-5B was transformed with pSAL4 [TP-Lac2] plasmid by the lithium acetate protocol (Gietz et al. 2002), the transformants were selected on minimal selective medium agar plates and the presence of the plasmid was confirmed by PCR on total DNA extracted from single clones. Positive colonies were picked, grown overnight in synthetic liquid medium (MM), and stored at − 80 °C as glycerol stocks. Cells from glycerol stock were subsequently inoculated overnight in fresh MM. Once in the exponential phase, cells were inoculated at OD_660_ 0.1 in MM supplemented with 0.3 mM CuSO_4_ and 0.1 M 2-(N-morpholino) ethanesulphonic acid - MES, at pH 6.0. Yeast cultures were incubated at 30 °C, at 160 rpm for 6 days (Cavallo s.r.l., Buccinasco, Italy). The cell growth and supernatant laccase activity were monitored by measuring the optical density at 660 nm and the oxidation of ABTS, respectively.

**Table 1:** List of primers used in this work. The *Eco*RI restriction site is underlined

Primer	Nucleotide sequence
Forward	GTACGAATTCAGATTATGTCGCGGTTCAAC
Reverse	CTGAATTCAGATCATGGTCGCTGGGGTC

### Purification of TP-Lac2

Culture supernatant was collected by sample centrifugation at 2000 g for 20 min and concentrated by tangential flow filtration with a Pellicon system (Millipore, Billerica, MA, USA), using 5-kDa cut-off filters. The retentate fraction (~ 70 mL), containing TP-Lac2, was frozen at − 80 °C and lyophilized for 72 h using a freeze-dryer (Cinquepascal s.r.l., Trezzano sul Naviglio, Italy). Lyophilized samples were dissolved in 10 mL of loading buffer (25 mM Tris–HCl, pH 8.0), filtered with a 0.22 μM filter (Euroclone, Pero, Italy). The samples were then purified by ion exchange chromatography using a DEAE-sepharose (Cytiva, MA, USA) manually impacted column, and an NGC Quest Plus Chromatography System (Bio-Rad, CA, USA). Samples were loaded on the pre-equilibrated column (25 mM Tris–HCl, pH 8.0) by using a sample pump at a flow rate of 0.1 mL/min. Proteins were subsequently purified with a NaCl gradient (0–1.0 M) at a flow rate of 0.75 mL/min. A total of 40 fractions were collected and assayed for laccase activity as described above. Fractions exhibiting the highest laccase activity were pooled together and buffer-exchanged with 20 mM ammonium acetate pH 7.0 by two consecutive gel filtrations on PD-10 columns (GE Healthcare, Little Chalfont, UK). Samples were lyophilized (Heto FD1.0, Gemini BV, Apeldoorn, the Netherlands) and stored at 4 °C. Protein concentration was determined by the Bradford protein assay, using bovine serum albumin as the standard. SDS-PAGE was on 14% acrylamide gels stained with Gel-Code Blue (Pierce, Rockford, IL, USA) after electrophoresis. Broad-range, pre-stained molecular-mass markers (GeneSpin, Milan, Italy) were used as standards.

### TP-Lac2 biochemical analysis

Lyophilized TP-Lac2 was suspended in sodium phosphate buffer (PB), 10 mM, pH 6.0 at a concentration of 1 mg/mL. All the reactions described in this paragraph were initiated by adding 5 mU of enzyme in 1 mL of reaction volume.

The temperature optimum (*T*_*opt*_) was determined within a temperature range of 15–75 °C using 25 mM tartrate buffer, pH 3.5 and 1 mM ABTS as a substrate. The pH optimum was examined in the pH range of 3.0–6.0 at 50 °C in 25 mM tartrate buffer.

Substrate specificity was determined at the optimal catalysis conditions with ABTS, 2,6-dimethoxyphenol (2,6 DMP), and guaiacol at a final concentration of 1 mM. Reactions were followed for 3 min at: 468 nm for 2,6-DMP (ε: 14.8 mM^−1^∙cm^−1^), 420 nm for ABTS (ε: 36 mM^−1^∙cm^−1^) and 465 nm for guaiacol (ε: 12 mM^−1^∙cm^−1^). Kinetic parameters were measured in 25 mM tartrate buffer, pH 3.5 at 50 °C, using different concentrations of ABTS (from 0.05 to 1.5 mM) and calculated with the ORIGINLAB software (OriginLab Corporation, Northampton, MA, USA), using the Michaelis–Menten equation.

The long-term thermal stability was monitored by measuring the residual enzymatic activity at optimal catalysis conditions after incubating TP-Lac2 at 30 °C and 50 °C for 5 days in PB at pH 6.0 or in tartrate buffer at pH 4.0. All the experiments were performed in quadruplicate and shown as mean ± standard deviation.

### TP-Lac2 structural analysis

The secondary structure of TP-Lac2 was studied by circular dichroism (CD) spectroscopy with a J815 spectropolarimeter (JASCO Europe, Cremella, Lecco). CD spectra (8 μM of TP-Lac2 in PB) were recorded using a 0.1 cm pathlength cuvette with a 0.2 nm data pitch and scanning speed of 20 nm∙min^−1^. All spectra were corrected for buffer contribution, averaged from three independent acquisitions, and smoothed by using a third order least-square polynomial fit. To study the thermal unfolding, we recorded the CD signal at a fixed wavelength of 215 nm in the temperature range 25–90 °C at a slope of 1 °C∙min^−1^.

The UV/visible absorption spectra (20 µM in PB) were collected with a V770 UV/NIR Jasco

V770 UV/NIR spectrophotometer (JASCO Europe, Cremella, Lecco) using a 1-cm pathlength quartz cuvette as described in Zampolli et al. (2023). The enzyme quaternary structure was investigated by SEC with a NGC Quest 10 Plus Chromatography System (Bio-Rad, CA, USA) equipped with a Superdex 10/100 column (Cytiva, MA, USA) with a cut-off of 10–75 kDa. Chromatographic separations were carried out in saline PB (10 mM PB, 150 mM NaCl, pH 6.0 - PBS) at a flow rate of 0.5 mL∙min^−1^ and a protein concentration of 0.5 mg/mL as described in Zampolli et al. (2023).

### Bioinformatics analysis

Sequence analysis and multiple sequence alignments of TP-Lac2 were carried out by using Clustal Omega (Sievers et al. 2011) and InterPro (Mitchell et al. 2019). The 3D model of TP-Lac2 was predicted with ColabFold (Mirdita et al. 2022), the Cu atoms were modelled by superimposing the 3D model of TP-Lac2 with the 3D structure of laccase from *Pycnoporus sanguineus* (PDB: 5nq7).

### Decolorization of synthetic dyes

Natural (AS, VAc and SA) and synthetic (ABTS, HBT) mediators were screened by monitoring the decolorization of five different dyes, namely amido black 10B, orange G, bromocresol purple sodium salt, erythrosin B and malachite green oxalate, with a Thermo Scientific Multiskan FC microplate reader (Waltham, MA, USA) in 96-well plates. The decolorization was monitored at the adsorption wavelengths listed in Table 2. The reaction mixtures contained TP-Lac2 (0.11 U/mL), dyes (50 mg/L), mediator (0.05 mM) and CuSO_4_ (0.5 mM), as suggested by Mandic et al. 2019, dissolved in 50 mM sodium tartrate buffer at pH 4.0. The plates were incubated on the 3400RVT orbital shaker (Cavallo s.r.l, Buccinasco, Italy) at 160 rpm for 3 h at 30 °C and 50 °C.

**Table 2:** Wavelength of absorption for specific dyes

Chemicals class	Dyes	λ_max_ (nm)	λ_tested_ (nm)
Azo dye	Amido black 10B	620	595
Azo dye	Orange G	479	492
Triphenylmethane dye	Bromocresol purple sodium salt	428	414
Di- and tri-aryl dye	Malachite green oxalate	618	595
Xanthene dye	Erythrosin B	529	540

The decolorization of amido black 10B, orange G, bromocresol purple sodium salt and malachite green oxalate was further investigated in the presence of different concentrations of AS and ABTS (0.025 mM and 0.05 mM) as mediators. Reactions were performed as previously described, incubated at 30 °C and 50 °C in an orbital shaker at 160 rpm for 24 h and the absorbance was measured after 0.5, 1, 3, 5 and 24 h. The decolorization rate was calculated using the following equation as described in (Si et al. 2013):

$${\text{Decolorization rate }}\left( \% \right) \, = { 1}00 \, * \, \left( {{\text{initial absorbance }}{-}{\text{ final absorbance}}} \right) \, /{\text{ initial absorbance}}$$

### Sequence availability

Both nucleotide and protein sequences for Tp-Lac2 have been deposited in GenBank (accession number OR828795).

## Results

### Identification of the filamentous fungi with the highest laccase activity

**Fig. 1:**
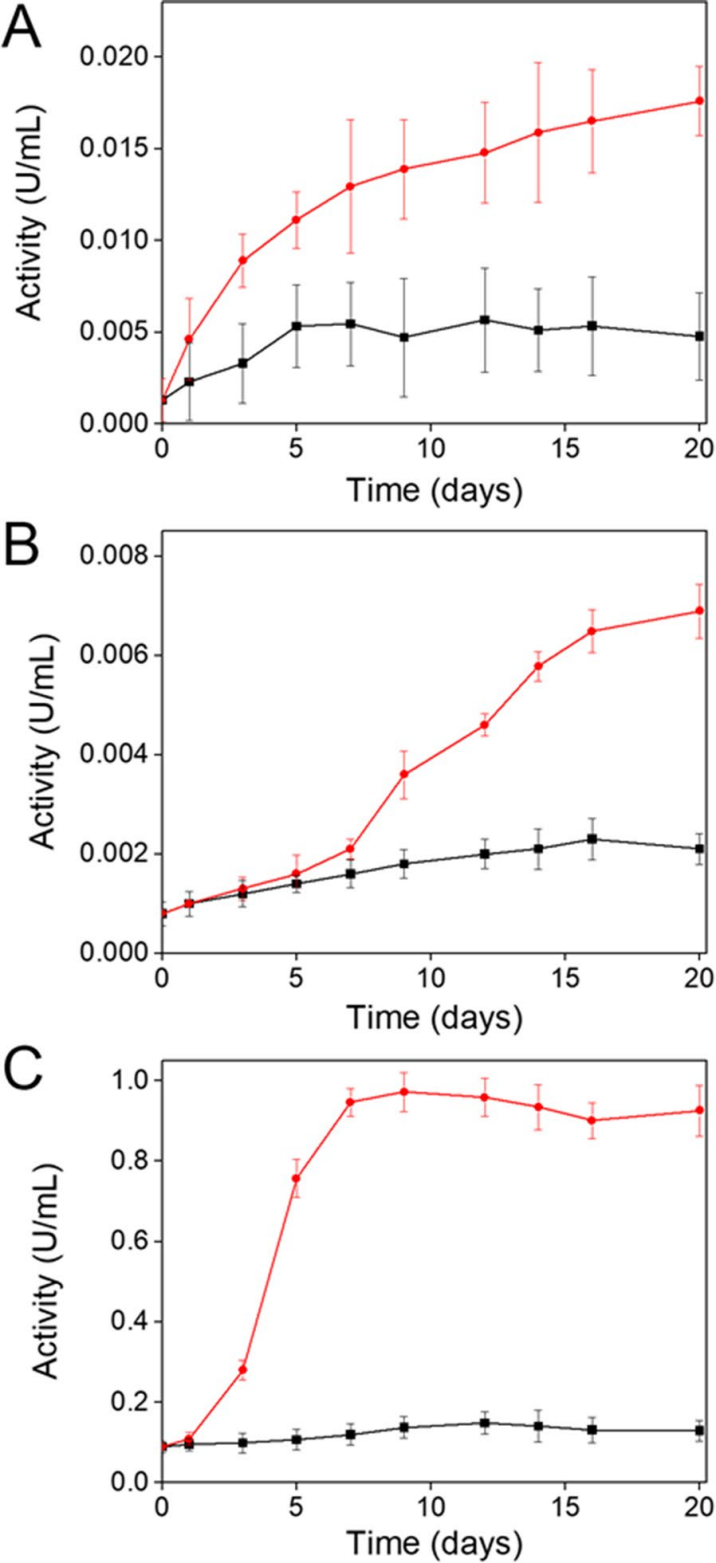
Identification of the best laccase producer. Liquid cultures of *H. annosum* (**A**), *S. ostrea* (**B**) and *T. polyzona* (**C**) were carried out on MM and the activity assay was performed on supernatants. The supernatants were periodically collected during the cultivations lasting 20 days in the absence (black lines) and in the presence of VA as inducer (red lines). Values are the mean ± standard deviation of three independent experiments. Note that the graphs in panels **A**, **B**, and **C** have different scales

A preliminary solid-plate screening of 6 white rot fungi (see Additional file 1) identified *T. polyzona*, *H. annosum* and *S. ostrea* ( Additional file 1:Fig. S1) as the best laccase producers. The potential laccase production of these strains was further investigated in liquid cultures on MM with and without VA, a known inducer of laccase expression (Viswanath et al. 2014). In all cases, in the absence of VA, laccase activity was very low and did not increase during cultivation (Fig. 1A–C). The addition of VA induced the laccase activities in all the three fungal cultivations. In particular, the highest activity was observed in the supernatant collected from the *T. polyzona* culture (Fig. 1C), which showed a 10-folds increase compared to the not induced one. In *T. polyzona* the highest activity value (0.97 U/mL) was reached after 9 days of cultivation, whereas the laccase activity measured in *H. annosum* and *S. ostrea* supernatants was much lower, in the presence of VA and after 20 days of cultivation, 0.018 U/mL and 0.007 U/mL, respectively (Fig. 1A, B).

To identify the enzyme(s) responsible for the high laccase activity, the total *T. polyzona* RNA was extracted, retro-transcribed and the cDNA was used as the template to amplify the putative laccase coding sequence, using primers designed based on the sequence of TP-Lac1. The sequence of the resulting amplicon was named TP-Lac2.

### Sequence and structural analysis of TP-Lac2

**Fig. 2:**
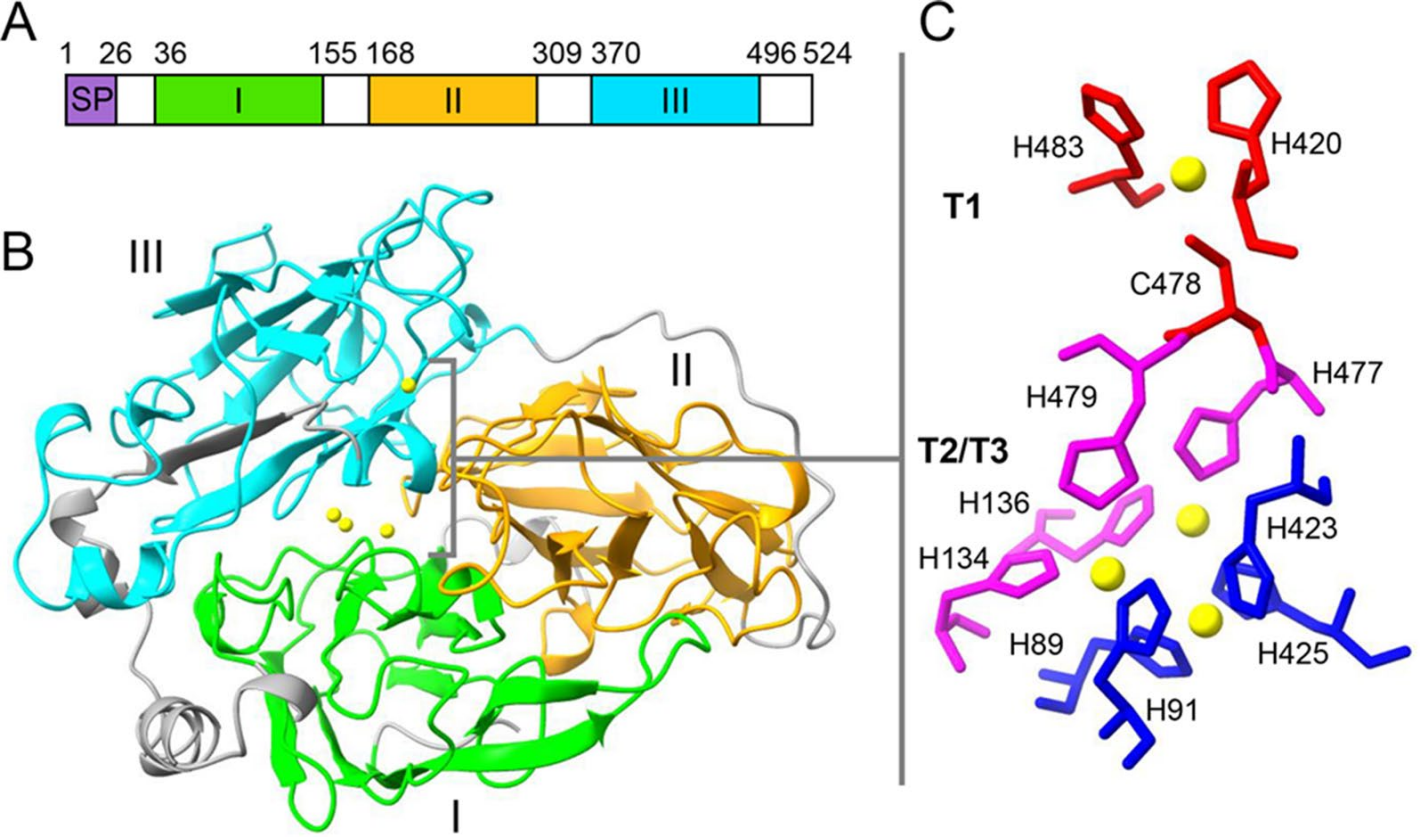
3D structure of TP-Lac2. **A** Architecture of TP-Lac2. **B** 3D model of TP-Lac2, domains I, II and III are colored in green, orange and cyan, Cu^2+^ ions are represented as yellow spheres. **C** T1, T2/T3 sites of TP-Lac2, residues of T1, T2 and T3 centers are represented red, blue and magenta sticks, respectively

**Fig. 3:**
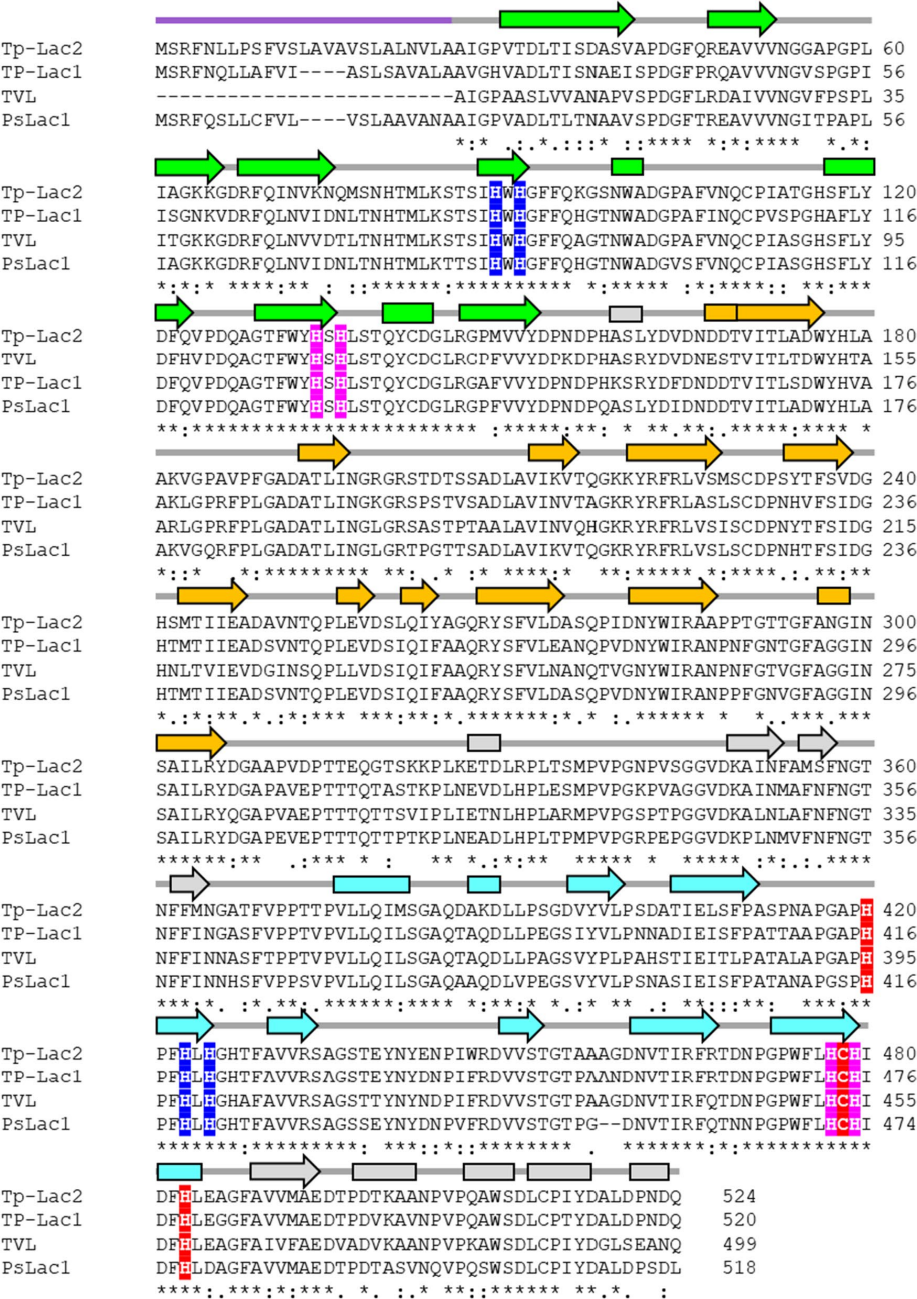
Sequence alignment of TP-Lac2 with its homologs. TP-Lac2 deduced amino acidic sequence is aligned with that of TP-Lac1 from the same fungus and with laccase from *P. sanguineus* isoform I (PSLacI, PDB: 5nq7) and from *T. versicolor* (TVL, PDB: 1GYC). Sequence alignment was carried out with Clustalw. Secondary structure elements extracted from PSLacI are shown at the top of the alignment (color code as in Fig. 2). Residues of T1, T2 and T3 centers are shaded in red, blue and magenta, respectively

Analyses of the computationally translated TP-Lac2 amino acid sequence indicates that this enzyme has the typical laccase architecture, consisting of three cupredoxin domains (Kumar et al. 2003; Hoegger et al. 2006), and harbours a signal peptide for secretion (Fig. 2A). TP-Lac2 shares 99% of amino acid sequence identity with the uncharacterized laccase/phenoloxidase from *T. polyzona* CIRM-BRFM 1798 [NCBI: KAI0637269.1 (Hage et al. 2021)], 77% of sequence identity with the TP-Lac1 [NCBI: AOZ19963.1 (Pi̇Nar et al. 2017)], 79 and 73.5% of sequence identity with the well characterized laccases from *Pycnoporus sanguineus* [PSLac1, PDB: 5NQ7, (Ramírez-Cavazos et al. 2014; Orlikowska et al. 2018)] and *Trametes versicolor* [TVL, PDB:1GYC, (Piontek et al. 2002)], respectively. The 3D model of TP-Lac2, predicted with Alphafold2 (via ColabFold (Mirdita et al. 2022)), shows the typical structure of laccase with a β-barrel containing three Greek key domains (Fig. 2B). The active site of laccases consists of a type 1 (T1) copper site that catalyzed the substrate oxidation, and a trinuclear copper cluster (T2 and T3 coppers) where the oxygen is reduced (Jones and Solomon 2015; Rovaletti et al. 2023). Residues involved in Cu ions coordination are conserved and identified by combining structural information and multiple sequence alignments (Figs. 2 and 3). In the T1 center, Cu ion is coordinated by H420, C478 and H483, while in the T2/T3 center Cu ions are coordinated by H89, H91, H423 and H425 (T2) and H91, H134, H136, H477 and H479 (T3) (Figs. 2C and 3).

### Functional and structural characterization of TP-Lac2

**Fig. 4:**
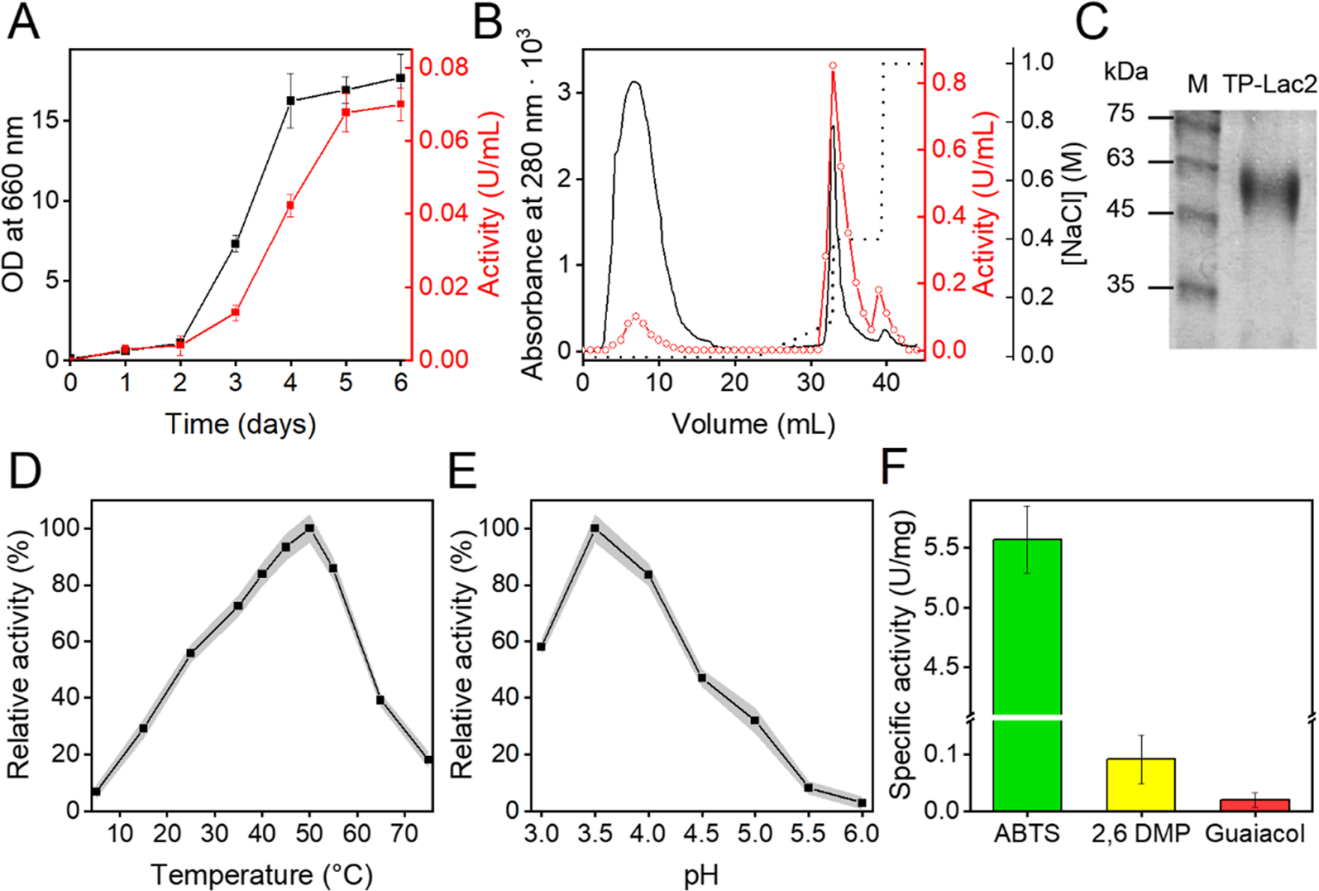
Recombinant production, purification and biochemical characterization of TP-Lac2. **A** Recombinant TP-Lac2 production in *S. cerevisiae* cells (CEN.PK Lac2): the laccase activity was assayed on supernatants of cultures at 25 °C, pH 4, by using ABTS as substrate. Experiments were performed in triplicate and the error bars indicate the standard deviation of the data. **B** Ion-exchange chromatography of culture supernatants on a DEAE-sepharose manually packed column: the laccase activity in the elution fractions (in red) was measured using the ABTS assay. The NaCl gradient is represented as a dotted line. **C** SDS-PAGE analysis of purified TP-Lac2. M: molecular weight marker. The effects of temperature (**D**) and pH (**E**) on the activity of TP-Lac2 were determined using ABTS as substrate (final concentration: 1 mM). Experiments were performed in quadruplicate and the shadowed area refers to the standard deviation of the data (n = 4). **F** Substrate specificity of TP-Lac2, assays were carried out using ABTS, 2,6-DMP and guaiacol in the optimal catalysis conditions at substrate concentration of 1 mM. Experiments were performed in quadruplicate and the error bars indicate the standard deviation of the data (n = 4)

The TP-Lac2 coding sequence, along with its own natural signal secreting sequence, was cloned into pSal4 plasmid under the control of copper-inducible *CUP1* promoter. Therefore, recombinant TP-Lac2 was produced in *S. cerevisiae* cells by inducing the heterologous expression with 0.3 mM CuSO_4_ at 30 °C for 6 days (Cassland et al. 1999; Pezzella et al. 2009). The addition of CuSO_4_ has the dual role of inducing transcription and enhancing enzyme activity by coordinating itself in the three cupredoxin domains (O’Callaghan et al. 2002; Liu et al. 2003). TP-Lac2 begins to accumulate in the culture supernatant after 3 days of incubation, during the exponential growth phase of *S. cerevisiae* cells: the maximum activity (0.07 U/mL) was observed after 6 days of incubation (Fig. 4A). It is worth mentioning that the recombinant enzyme was efficiently secreted by *S. cerevisiae* cells thanks to its native secretion sequence, as confirmed by the poor intracellular laccase activity that accounted for less than 10% of the total laccase activity checked at every time points (data not shown).

TP-Lac2 was purified to homogeneity from the culture supernatant by ion exchange chromatography (Fig. 4B, C). The SDS-PAGE analysis showed a band with a molecular weight of ~ 60 kDa, accompanied by a smear typical of glycosylated proteins (Fig. 4C). The glycosylation of TP-Lac2 was also confirmed through deglycosylation experiments (data not shown). At the end of purification, the TP-Lac2 specific activity was 4.83 ± 0.45 U/mg on ABTS at 25 °C. The effects of temperature and pH on TP-Lac2 activity were investigated using ABTS as substrate. TP-Lac2 maintains high activity at acidic pH and in the temperature range 35–60 °C with an optimal temperature (*T*_*opt*_) of 50 °C and pH of 3.5 (Fig. 4D, E), among those tested. The substrate specificity of TP-Lac2 was studied on phenolic (2,6 DMP and guaiacol) and non-phenolic (ABTS) compounds at optimal catalysis conditions. TP-Lac2 is able to oxidize all the tested compounds with the highest activity detected on ABTS (Fig. 4F). The kinetics parameters of TP-Lac2 display a *K*_*M*_ of 0.25 ± 0.04 mM and a *V*_*max*_ of 2.02 ± 0.12 mM/min in the presence of ABTS.

**Fig. 5:**
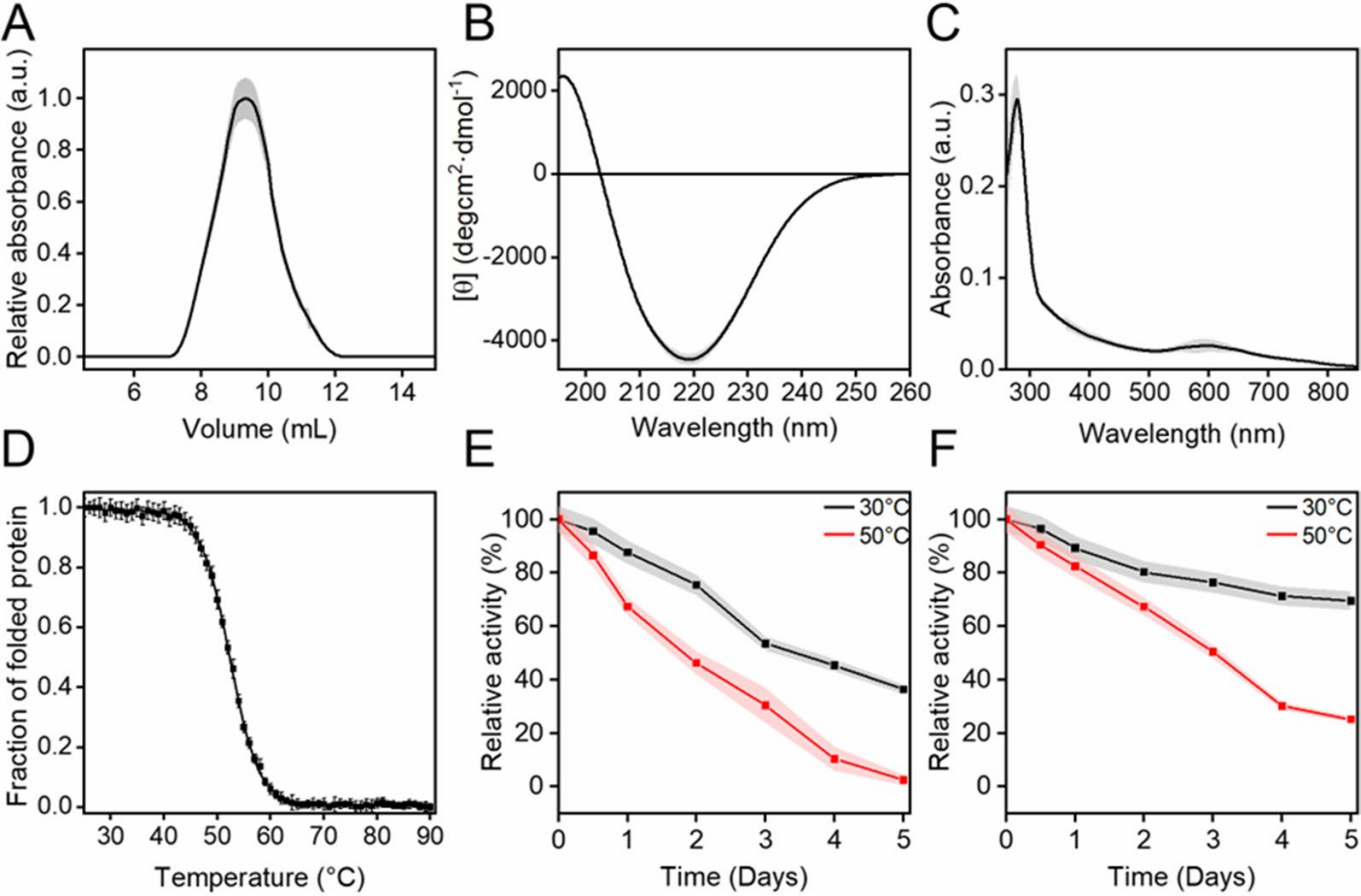
Biophysical characterization and thermal stability of TP-Lac2. **A** Quaternary structure of TP-Lac2 determined by SEC chromatography in PBS at 25 °C. **B** CD spectrum of TP-Lac2 recorded in PB at 25 °C. **C** UV/Vis absorption spectrum of TP-Lac2 collected in PB at 25 °C. **D** Thermal stability of TP-Lac2. Ellipticity values were recorded at 218 nm during heating from 25 to 90 °C. The initial CD signal was taken as 100% for normalization. Long-term thermal stability of TP-Lac2 at pH 4.0 (**E**) and 6.0 (**F**). Thermal stability over time was measured by incubating the enzyme at 30 °C and 50 °C and shown as residual activity. All the experiments were performed in triplicate and the shadowed area refers to the standard deviation of the data (n = 3)

SEC analysis indicates that TP-Lac2 is a monomer (Fig. 5A, molecular mass: 60.1 kDa) with a secondary structure enriched in β-sheets as indicated by the CD spectrum, which is characterized by a minimum peak at ~ 219 nm (Fig. 5B). Moreover, TP-Lac2 is a blue laccase as indicated by the peak at ~ 608 nm (Fig. 5C), which is typical of the paramagnetic T1 center (Jones and Solomon 2015). Overall, these structural data are consistent with the predicted 3D model previously described. The unfolding transition midpoint (*T*_*M*_), determined by CD analysis in the temperature range of 25–90 °C, is 53.4 ± 0.85 °C, indicating that TP-Lac2 displays a good thermal stability (Fig. 5D). To deeper investigate the TP-Lac2 robustness, we tested thermal stability at two temperatures (30 and 50 °C) and two different pH values (4.0 and 6.0). At pH 4.0, we observed a sharp decrease of TP-Lac2 activity at both temperatures, with greater inactivation at 50 °C (Fig. 5E). At pH 6.0, TP-Lac2 is more stable than at pH 4.0, with 69.2 and 26.7% residual activity after 5 days at 30 and 50 °C (Fig. 5F).

### Dye decolorization assay with five mediators

**Fig. 6:**
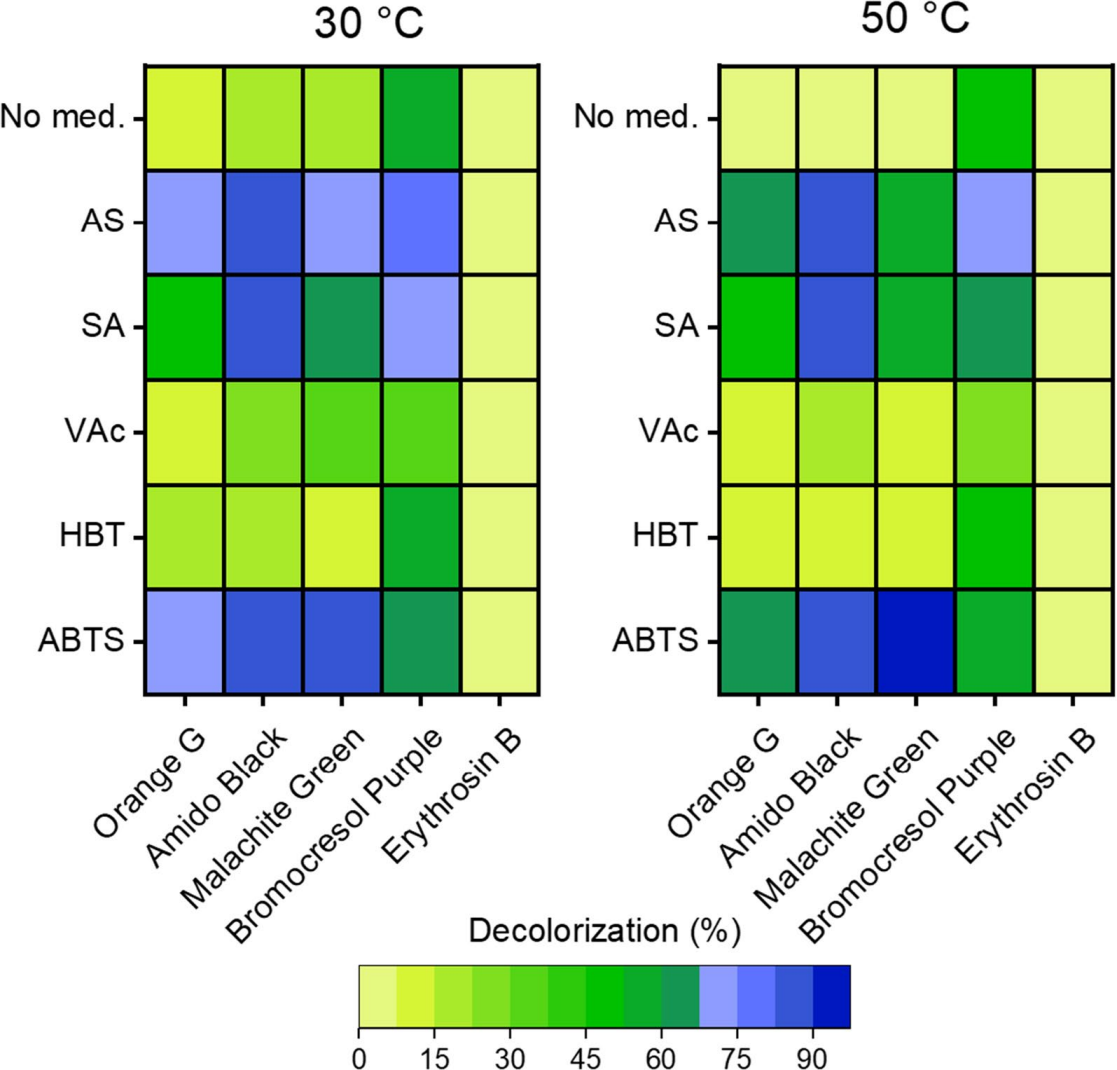
Ability of TP-Lac2 to decolorize different synthetic dyes in the presence of different mediators. The reactions were carried out in 96- well plates at 30 °C and 50 °C for 3 h with 0.05 mM ABTS, Benzothiazole (HBT), Acetosyringone (AS), Vanillic acid (VAc) and Syringaldehyde (SA). Experiments were performed in triplicate

The ability of TP-Lac2 to decolorize synthetic dyes was investigated on five different textile dyes: two azo dyes (amido black 10B and orange G) (Selvam et al. 2003), bromocresol sodium salt, which is a triphenylmethane dyes (Gessner and Mayer 2000), the di- and triaryl dye malachite green oxalate (Sun et al. 2021) and the erythrosin B, which belongs to the xanthene class (Mandic et al. 2019). Tests were performed either in the absence and in the presence of 0.05 mM of synthetic (ABTS and HBT) and natural (VAc, SA and AS) mediators. Reactions were performed at 30 °C and 50 °C and the decolorization yield was evaluated after 3 h of incubation. In the absence of mediators, TP-Lac2 partially oxidizes the tested dyes (decolorization rate < 15%), with the exception of bromocresol purple sodium salt, which is oxidized with a decolorization rate of ~ 50% at both 30 °C and 50 °C (Fig. 6). In the presence of mediators, the only dye that is not oxidized was erythrosin B (Fig. 6). At both temperatures the most effective mediators are AS and ABTS with dye decolorization rates higher than 65%, while in the presence of VAc and HBT the decolorization rates are similar to those observed without mediators (Fig. 6). An atypical behaviour was observed with SA: the decolorization rates in the presence of amido black 10B and bromocresol purple sodium salt are higher than 80%, while with orange G and malachite green oxalate the decolorization rates are ~ 50% (Fig. 6).

**Fig. 7:**
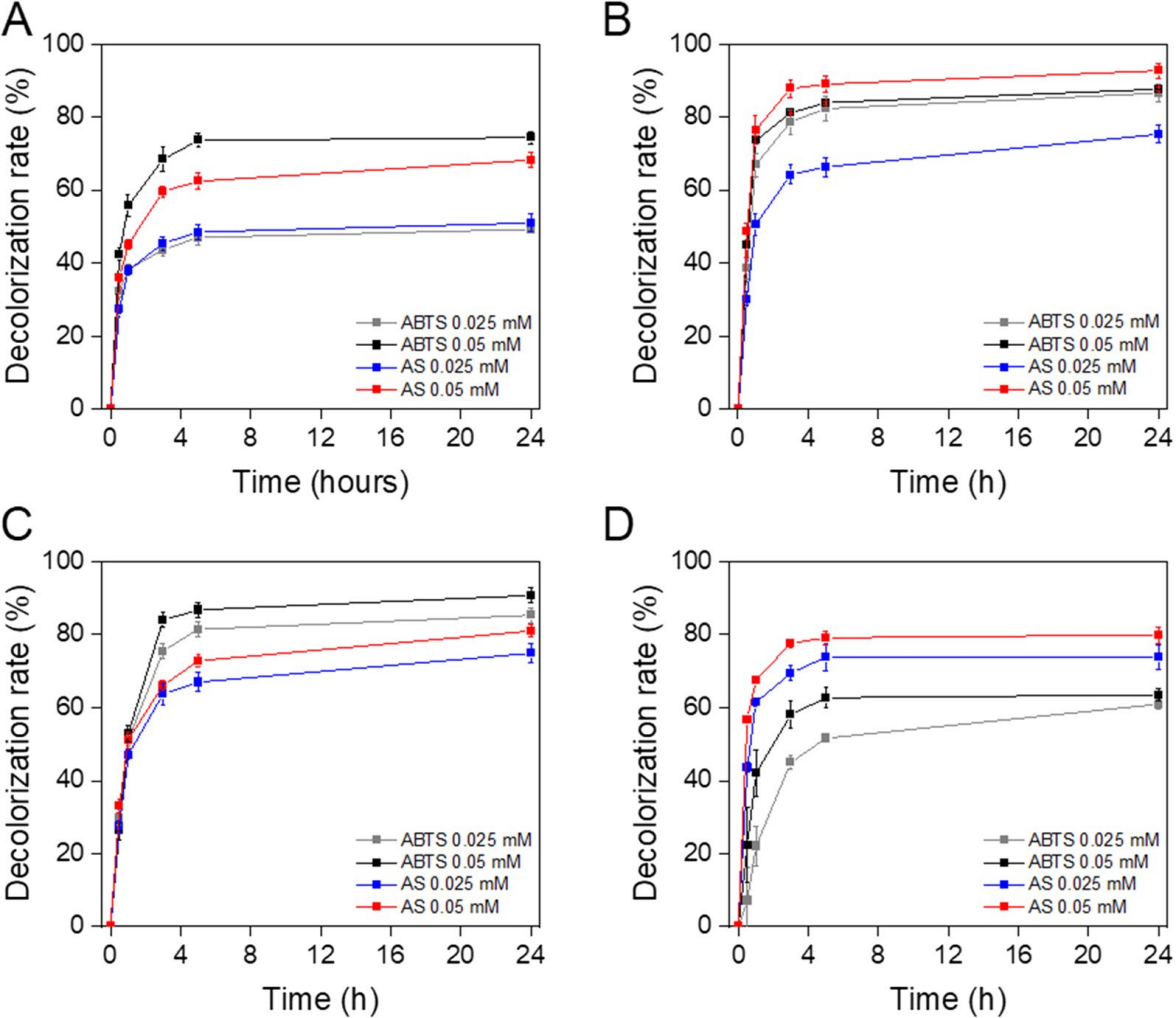
Kinetic of decolorization of synthetic dyes at 30 °C. TP-LAC2 was incubated in the presence of orange G (**A**), amido black 10B (**B**), malachite green oxalate (**C**) and bromocresol purple sodium salt (**D**) with ABTS and AS for 24 h at 30 °C. The decolorization rate was determined as the mean ± standard deviation of three independent experiments

**Fig. 8:**
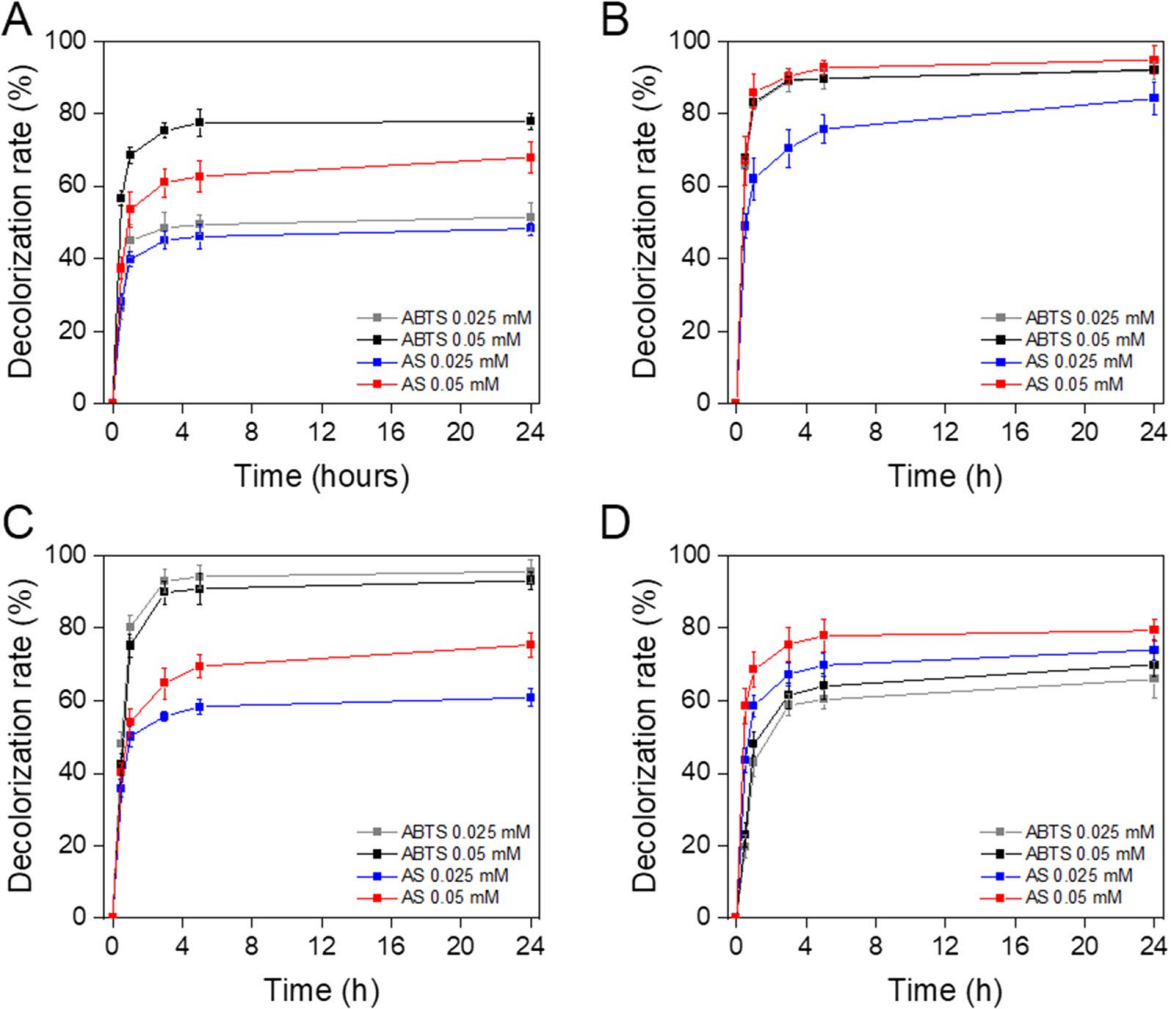
Kinetic of decolorization of synthetic dyes at 50 °C. TP-LAC2 was incubated in the presence of orange G (**A**), amido black 10B (**B**), malachite green oxalate (**C**) and bromocresol purple sodium salt (**D**) with ABTS and AS for 24 h at 50 °C. The decolorization rate was determined as the mean ± standard deviation of three independent experiments

The ability of TP-Lac2 to decolorize synthetic dyes was studied more in depth by measuring the decolorization rate over time at 30 °C and 50 °C in the presence of the two best mediators (i.e. AS and ABTS) at two different concentrations (0.025 mM and 0.05 mM). At 30 °C, all the reactions reached a plateau within 5 h of incubation with a different extent, depending on the dye tested (Fig. 7). In most cases, the best results are obtained at the highest concentrations of both mediators (Fig. 7), with AS being most active on amido black 10B (92.8 ± 2.4% after 24 h) and bromocresol purple sodium salt (79.8 ± 2.3% after 24 h), and ABTS on orange G (72.3 ± 1.7% after 24 h) and malachite green oxalate (90.6 ± 3.2% after 24 h). Similar results with faster kinetics were observed at 50 °C (Fig. 8).

Specifically, AS was most effective on amido black10B (94.6 ± 0.8% after 5 h) and bromocresol purple sodium salt (72.2 ± 0.8% after 5 h) whereas ABTS on orange G (79.3 ± 0.9% after 5 h) and malachite green oxalate (94.6 ± 0.1% after 5 h).

## Discussion

Although the ability of laccases from white-rot fungi to decolorize dyes has been widely reported, the search for new laccases is motivated by the huge repertoire of synthetic dyes used by the textile industry (Erkurt et al. 2007; Fillat et al. 2012; Zheng et al. 2017; Ancona-Escalante et al. 2018; Kumar and Chandra 2020; Eichlerová and Baldrian 2020). In the search for novel laccases for textile dyes decolorization, we screened six different white-rot fungi known for their ability to grow on lignocellulosic biomass. Among these, *T. polyzona* was shown to be the best laccase producer. This is not surprising given that several species within *Trametes* genus, belonging to the *Polyporaceae* family (Dai 2012), have been reported to produce laccases at high levels (Zheng et al. 2017). Although several biotechnological applications have been proposed for crude *T. polyzona* laccases (Jaouani et al. 2006; Cabana et al. 2007, 2009), only two laccases (i.e. TP-Lac1 and *Tp*L) from this fungus have been partially characterized (Pi̇Nar et al. 2017; Ezike et al. 2020)*.* The latter are produced by *T. polyzona* in minimal medium without the addition of any inducers and exhibit similar biochemical features (Pi̇Nar et al. 2017; Ezike et al. 2020).

In this study, when *T. polyzona* is cultivated in minimal medium, the assayed laccase activity is low and increases in the presence of VA, a xenobiotic compound known to induce the activity of laccases in white-rot fungi (Viswanath et al. 2014). In the presence of this inducer, the strain of *T. polyzona* secretes what we identified as TP-Lac2, which shares 77% sequence identity with TP-Lac1 and exhibits different substrate specificity, *T*_*opt*_ and temperature stability compared to TP-Lac1 and *Tp*L. Our results support the previous evidences showing that the production of various isoenzymes with different catalytic properties is a strategy of *T. polyzona* to adapt in different environments or to respond to different stimuli (Giatti Marques De Souza et al. 2004; Dittmer et al. 2006; Yuan et al. 2016). To better characterize this novel enzyme, its full coding sequence was expressed in *S. cerevisiae*, a yeast that lacks a corresponding enzymatic activity. The laccase activity of recombinant TP-Lac2 (0.07 ± 0.01 U/mL) is lower than that observed in the medium of *T. polyzona* cultures (0.92 ± 0.06 U/mL). This difference may be attributed to the hyperglycosylation of Tp-Lac2 in yeast cells, as reported in other recombinant fungal laccases (Garg et al. 2012; Maestre-Reyna et al. 2015). We demonstrated that TP-Lac2 is active in a wide range of temperatures and is thermostable, as it retains more than 50% of its initial activity after 2 days of incubation at 50 °C and pH 6.0. Overall, these features make TP-Lac2 a suitable candidate for bioremediation applications, such as decolorization of textile dyes, in uncontrolled open environments where factors, such as temperature, are difficult to control (Viswanath et al. 2014).

Numerous examples can be found in the literature where white-rot fungi species including the genus of *Trametes* have been reported to produce laccases with the ability to decolorize various dyes: some examples are *Pleurotus ostreatus*, *Trametes versicolor*, *Funalia trogii*, *Trametes hirsuta*, *Trametes villosa, T. polyzona,* and *Trametes orientalis* (Erkurt et al. 2007; Fillat et al. 2012; Zheng et al. 2017; Ancona-Escalante et al. 2018; Kumar and Chandra 2020; Kumar and Chandra 2020; Eichlerová and Baldrian 2020).

In the absence of mediators, TP-Lac2 alone is unable to decolorize almost all the dyes tested (decolorization rate <15%), except for bromocresol purple sodium salt (~ 50%), probably due to its aromatic structure or redox potential. However, when using the mediators ABTS and AS, TP-Lac2 was able to decolorize four of the dyes tested, namely malachite green oxalate, amido black 10B, orange G, and bromocresol purple sodium salt, with yields comparable to or higher than those reported for other laccases. Using the mediators ABTS and AS, TP-Lac2 achieved a decolorization rate of 94.6% in just 5 h at 50 °C with malachite green oxalate and amido black 10B. This rate is significantly higher than that of *Tp*L laccase from *T. polyzona* WRF303, which had a decolorization rate of malachite green oxalate of 57.8% (Ezike et al. 2020), and similar to that of laccase isolated from the litter-decomposing fungus *Gymnopus luxurians* with AS as a mediator (Sun et al. 2021).

Remarkably, the Tp-Lac2 demonstrated an impressive ability to degrade orange G, a recalcitrant azo dye (Selvam et al. 2003; Eichlerová and Baldrian 2020), with a degradation rate of 79.3% when ABTS was added to the cocktail. This is a significant finding as laccases from *Pseudomonas* species were previously reported to be unable to degrade such dyes (Mandic et al. 2019). In addition, the laccase from the white-rot fungus *Thelephora* sp, which was used for synthetic dye decolorization, failed to achieve more than 50% decolorization of orange G (33.3%) (Selvam et al. 2003). This phenomenon might be attributed to the fact that both laccases (from *Pseudomonas* sp. and *Thelephora* sp.) were employed without the addition of mediators.

The use of mediators is attractive in the context of bioremediation to reduce the toxicity and cost of the process (Camarero et al. 2005; Cañas and Camarero 2010; Fillat et al. 2012; Ancona-Escalante et al. 2018). However, there are drawbacks associated with the use of synthetic redox mediators such as ABTS, TEMPO, and HBT because they are expensive and proved to be toxic (Hu et al. 2009). Our study showed that the LMS consisting of Tp-Lac2 and AS is comparable to that of ABTS. AS is a natural mediator as it can be derived from lignin degradation or oxidation (Camarero et al. 2005; Cañas and Camarero 2010). Our results confirm previous findings that natural mediators with two methoxy groups, such as AS and SA, are more efficient in the decolorization reaction than VAc, which has a single methoxy group (Fillat et al. 2012; Park et al. 2021). It is noteworthy that the concentration of AS used in our LMS is less than 1 mM (0.05 mM), a concentration at which this mediator did not significantly inhibit melanoma cell viability as reported by Park et al. 2021. Overall, the decolorization results obtained with a natural mediator such as AS are promising in view of its industrial applications. Furthermore, the difference in molecular weight should not be underestimated. Specifically, ABTS exhibits a molecular weight that is approximately 2.5 times higher than the one of AS. Consequently, in a prospective industrial application, a lower quantity of mediator would be required to obtain the same number of moles if AS is used.

In conclusion, the biochemical characterization of TP-Lac2 indicates that it is indeed a non-characterized new laccase that differs from those already described in literature.

This enzyme can be used in the presence of synthetic and natural mediators: the mediator system consisting of TP-Lac2 and AS holds potential as an environmentally friendly alternative for wastewater treatment in the textile industry, replacing chemical-based methods. The feasibility of the heterologous production has been demonstrated, yet further improvements could be achieved by altering for instance the secretory sequence or by optimizing the codon usage or by fine-tuning the expression level, as reported previously (Antošová and Sychrová 2016).

### Supplementary Information

**Additional file 1: Figure S1** Solid-plate screening of fungi for laccase production. Fungal species were grown at 30 °C for 7 days on MEA plates supplemented with 3 mM ABTS to identify putative laccase activity.

## Data Availability

The datasets generated during the current study are available from the corresponding author on reasonable request.

## Abbreviations

ABTS: 2,2′-Azinobis (3-ethylbenzthiazoline-6-sulfonate)
HBT: 2-(2-hydroxyphenyl) benzothiazole
2,6 DMP: 2,6-Dimethoxyphenol
AS: Acetosyringone
LMS: Laccase/mediator system
MM: Minimal medium
PB: Sodium phosphate buffer
SA: Syringaldehyde
TP-Lac1: *Trametes polyzona* laccase isoform 1
TP-Lac2: *Trametes polyzona* laccase isoform 2
Vac: Vanillic acid
VA: Veratryl alcohol
