# Supplementary material for: A novel laccase from Trametes polyzona with high performance in the decolorization of textile dyes
Source: AMB Express. 2024 Mar 20;14:32. doi: 10.1186/s13568-024-01687-3 (PMC10954600; doi:10.1186/s13568-024-01687-3)
Supplement: Supplementary file 1 — Additional file 1: Figure S1 Solid-plate screening of fungi for laccase production. Fungal species were grown at 30 °C for 7 days on MEA plates supplemented with 3 mM ABTS to identify putative laccase activity. [file 13568_2024_1687_MOESM1_ESM.docx]

Additional file Materials for

**A novel laccase from *Trametes polyzona* with high performance in the decolorization of textile dyes**

Daniela Bucchieri ^1,2, $^, Marco Mangiagalli ^1, $^, Francesca Martani ^1^, Pietro Butti ^1^, Marina Lotti ^1^, Immacolata Serra *****^1^, Paola Branduardi ^1^

^1^ Department of Biotechnology and Biosciences, University of Milano-Bicocca Piazza della Scienza 2, 20126, Milano, Italy

^2^ Department of Material Science and Nanotechnology, CORIMAV program, University of Milano-Bicocca Via R. Cozzi 55, 20125, Milano, Italy

^$^ contributed equally to this work

***Corresponding author: immacolata.serra@unimib.it**

**Solid-plate screening of filamentous fungi to identify laccase activity**

Six white-rot fungi, i.e. *Armilaria mellea*, *Trametes polyzona*, *Deadaleopsis confragosa*, *Heterobasidion annosum*, *Stereum hirsutum* and *Stereum ostrea*, were screened on MEA plates supplemented with ABTS, a known chromogenic laccase substrate (Senthivelan et al. 2019). A heat-sterilized scalpel was used to cut from the agar plate a 1 cm² square of the mycelia that was transferred in new sterile malt extract agar (MEA) plates containing 3 mM ABTS. The plates were observed for green color development to screen for laccase activity as described in (Senthivelan et al. 2019). After 7 days of incubation at 30 °C, green zones, which indicate ABTS oxidation, were observed around all strains (**Fig. S1**). Higher growth and uniform green activity staining under the mycelium largest green zones were observed for *T. polyzona*, *H. annosum* and *S. ostrea.*


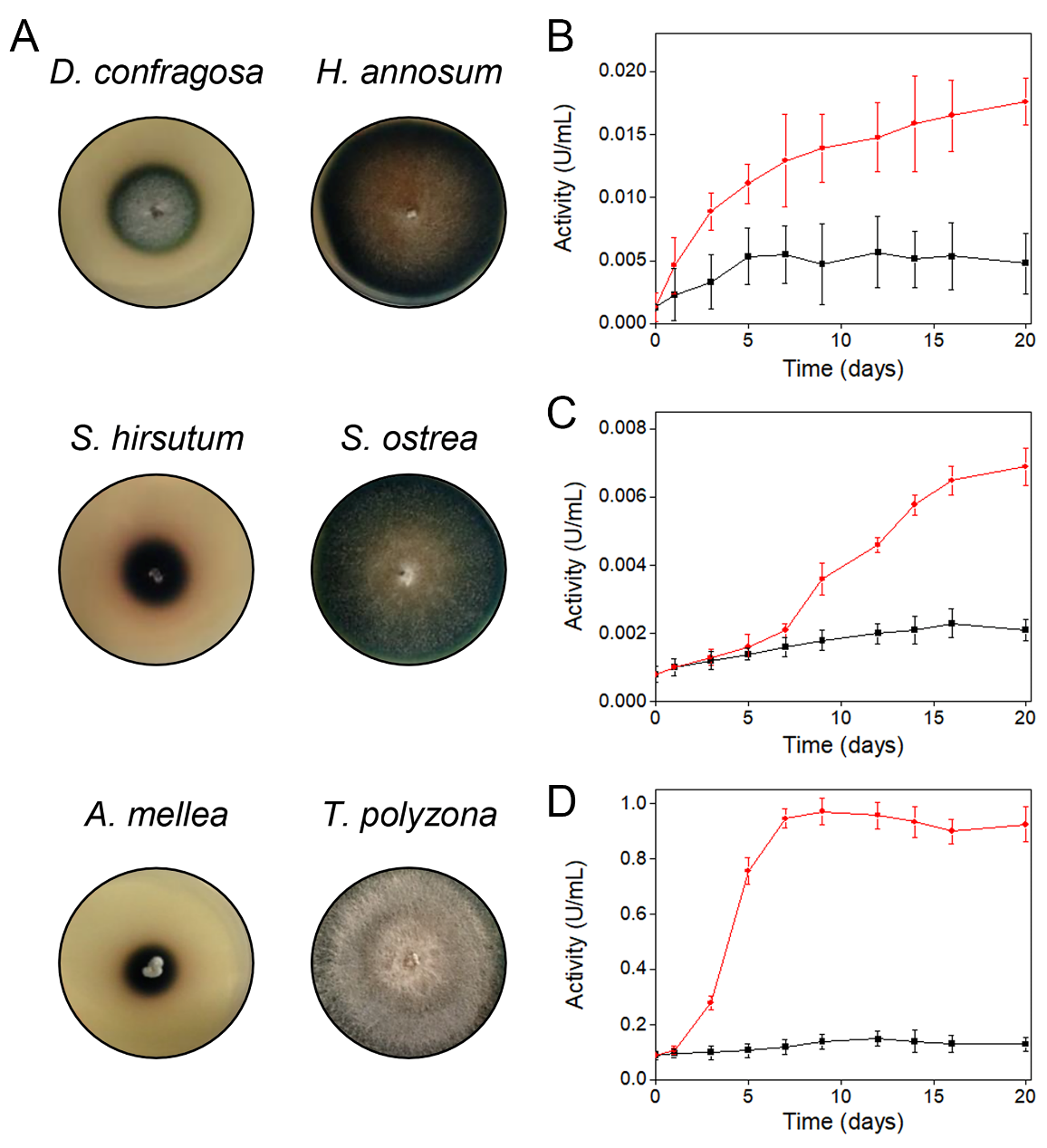


**Figure S1. Solid-plate screening of fungi for laccase production.** Fungal species were grown at 30°C for 7 days on MEA plates supplemented with 3 mM ABTS to identify putative laccase activity.
